# Neuromagnetic representation of musical roundness in chord progressions

**DOI:** 10.3389/fnins.2024.1383554

**Published:** 2024-04-08

**Authors:** Sophie D. Wöhrle, Christoph Reuter, André Rupp, Martin Andermann

**Affiliations:** ^1^Section of Biomagnetism, Department of Neurology, Heidelberg University Hospital, Heidelberg, Germany; ^2^Musicological Department (Acoustics/Music Psychology), University of Vienna, Vienna, Austria

**Keywords:** MEG, auditory evoked fields, musicality, dissonance, roundness

## Abstract

**Introduction:**

Musical roundness perception relies on consonance/dissonance within a rule-based harmonic context, but also on individual characteristics of the listener. The present work tackles these aspects in a combined psychoacoustic and neurophysiological study, taking into account participant’s musical aptitude.

**Methods:**

Our paradigm employed cadence-like four-chord progressions, based on Western music theory. Chord progressions comprised naturalistic and artificial sounds; moreover, their single chords varied regarding consonance/dissonance and harmonic function. Thirty participants listened to the chord progressions while their cortical activity was measured with magnetoencephalography; afterwards, they rated the individual chord progressions with respect to their perceived roundness.

**Results:**

Roundness ratings differed according to the degree of dissonance in the dominant chord at the progression’s third position; this effect was pronounced in listeners with high musical aptitude. Interestingly, a corresponding pattern occurred in the neuromagnetic N1m response to the *fourth* chord (i.e., at the progression’s resolution), again with somewhat stronger differentiation among musical listeners. The N1m magnitude seemed to increase during chord progressions that were considered particularly round, with the maximum difference after the final chord; here, however, the musical aptitude effect just missed significance.

**Discussion:**

The roundness of chord progressions is reflected in participant’s psychoacoustic ratings and in their transient cortical activity, with stronger differentiation among listeners with high musical aptitude. The concept of roundness might help to reframe consonance/dissonance to a more holistic, gestalt-like understanding that covers chord relations in Western music.

## 1 Introduction

Music has mattered to mankind for thousands of years. At the same time, music perception is highly subjective, and preferences differ whether, e.g., something sounds “suitable” or “cohesive” or “round.” Regarding harmony, the perception of musical roundness is based on at least three aspects: (1) Consonance/dissonance, (2) the musical context, and (3) the individual background of the listener. In this paper, we report a magnetoencephalography (MEG) experiment that is designed to jointly take these aspects into account. Specifically, psychoacoustic roundness ratings and the musical aptitude of our participants were matched with their neuromagnetic response to chord progressions in which dissonance was varied based on a well-established context of Western music theory.

Consonant and dissonant intervals and chords characterize western music and are debated since ancient times. If two or more sounds are played at the same time, they are commonly judged as pleasant (= consonant) or disturbing (= dissonant). Pythagoras linked this to the simplicity of the interval’s frequency ratios, e.g., 1:2 in the octave or 2:3 in the fifth (Dahlhaus, 2016, ch. 2).

In his seminal work, Helmholtz (1863, 1878) laid the foundations for the consonance theories we use today: On the one hand, he defined the consonance of intervals as the absence of roughness or beats (Helmholtz, 1863, p. 275ff.). Helmholtz’s “roughness curve” showed a ranking from the most consonant to the most dissonant interval. This ranking is also the result of almost all consonance theories known today. On the other hand, in the second part of his work, Helmholtz presented a difference-tone theory for the consonance of chords (Helmholtz, 1863, p. 320ff.): According to this theory, those chords are perceived as most consonant whose first and second order difference tones [i.e., the quadratic (D11) and cubic difference tone (D21), (Zwicker and Fastl, 2007, p. 277ff.)] correspond to pitches that are already contained within the chord. Based on these two approaches, three types of consonance theories emerged in the course of the 20th century:

1. *The absence of roughness or beats: The less roughness or beats an interval has, the more consonant the sound is perceived to be*. Although Plomp and Levelt as well as Kameoka and Kuriyagawa could pursue this theory (Plomp, 1964; Plomp and Levelt, 1965; Kameoka and Kuriyagawa, 1969a,b), it is now generally disproved: If the interval components are presented dichotically and therefore cannot interfere in the cochlea, the interval is still perceived as dissonant (Bidelman and Krishnan, 2009; McDermott et al., 2010).
2. *Difference tone theories: The more the quadratic and cubic difference tones from the partials of a chord or interval enter into an octave or fifth relationship with one of the chord tones, the more consonant the sound is perceived to be*. Krueger (1903, 1906) and Sandig (1938) expanded this theory into a system for all intervals within an octave. Paul Hindemith adopted this system in his “Unterweisung im Tonsatz” (Hindemith, 1938, 1945), which made it very popular for the explanation of musical consonance and dissonance. Husmann (1953) showed that the perception of consonance is disturbed when pure tones are presented binaurally, while consonance perception works very well when the binaurally presented sounds have partials. In other words, consonances and dissonances can be perceived even when there are no difference tones between the partials of two interval tones. It is only in the case of pure tones that the perception of consonance and dissonance becomes more difficult. This supports the coincidence theories:
3. *Coincidence theories: The more the partials of two sounds of an interval coincide, the more consonant the sound is perceived to be*. Only a few decades after Helmholtz, Carl Stumpf established his understanding of consonance as the fusion of sounds, which he revealed by studying “non-musical people” (Stumpf, 1890a,b). Although Stumpf considered the partials’ coincidence as a by-product rather than a reason for consonance perception, the explanatory approach of coinciding partials provides a serious alternative to the theory of roughness/beats or difference tones. The advantage of this approach is that it can be applied both in the frequency domain (for coincident partials) and in the time domain (for coincident periods), describing essentially the same phenomenon. From a frequency domain perspective, which he referred to as sensory consonance, Ernst Terhardt (1972) derivates the harmony perception from coinciding subharmonics in his virtual pitch model. Here, subharmonics are calculated from the first 8 (later 6) partials of an incomplete sound, and at the point where most of the subharmonics coincide the frequency of the virtual pitch or residue can be found (Terhardt, 1972). While for consonant intervals the point with the most coincidences falls on a pitch already contained in the interval, for dissonant intervals no unique virtual pitch can be determined (Terhardt, 1976; Terhardt, 1984). The coincidences of virtual subharmonics postulated by Terhardt are mirrored in the time domain by the neuronal interspike interval (ISI) distributions described by Tramo et al. (2001): Here, too, there is a periodic pattern within a time window of 50 ms for consonant intervals, whereas no or less clear pattern can be seen for dissonant intervals (siminlar in Ebeling, 2007; Ebeling, 2008). Parncutt and Hair (2011) suggested that Terhardt’s concept was misleading and introduced a novel synthesis of music theory, psychoacoustics, and dichotomies such as tense/relaxed, familiar/unfamiliar, rough/smooth, fused/segregated, and so on. Nevertheless, Terhardt’s understanding of consonance perception has influenced many studies (Bigand et al., 1996; Minati et al., 2009; McLachlan et al., 2013), and – supported by the time domain perspective (autocorrelation) in the field of neural processing (Tramo et al., 2001, see above) – this approach is also the basis of the current experiment.

The tonal center of a musical context is the tonic (Jones, 1974; Stainer, 2009), a chord of three tones in thirds (e.g., *c – e – g*). It is built on the first tone of a scale. Every other chord is related to it and leads more or less back to the tonic. One example is the subdominant, which is formed on the fourth tone of a scale (*f – a – c – in C major*) and is often used to extend the musical context. The dominant which starts on the fifth tone of a scale (*g – b – d in C major*) creates some tension and has the strong tendency to lead back to the tonic. Dominant chords can be modified by adding several tones in and out of key. One famous example is the dominant seventh chord with its additional flat seventh (*g – b – d – f in C major*). Ending a melody or chord progression with a dominant chord leaves the listener with a disturbing feeling of incompleteness. A chord can have different functions in different musical contexts, even the most consonant chord can be disturbing if it is placed within the ‘wrong’ context. Experiments on consonance/dissonance should therefore rely on chord progressions rather than single, isolated chords. In our study, we use the term “roundness” in order to avoid confusion with the long-grown terms “consonance” and “dissonance” and to capture not only the perception of single chords but also that of chord progressions. Roundness can be understood as a form of closure or gestalt. How well do chords fit together? Is the chord progression cohesive? Unlike consonance and dissonance, roundness is more natural in its valence and offers a broader range of subjective perceptions. Up to date, roundness has no explicit definition; yet, the psychoacoustic results will demonstrate that our listeners had a homogeneous understanding of the concept.

Previous studies on consonance and dissonance have examined neural responses at the brainstem and the cortical level. The neuronal pitch salience (NPS), measured as frequency following response (FFR) in the brainstem, correlates with musicological conceptions of consonance and dissonance. Amplitudes are larger and latencies shorter for consonant dyads than dissonant ones (Bidelman and Krishnan, 2009; Bidelman, 2013; Bidelman and Grall, 2014). Bidelman and Krishnan (2011) extended this result to different chords that are often used in western classical music (major, minor, augmented, diminished); here, their frequency of occurrence in compositions correlated was reflected in the FFR. Those findings are, however, limited by the fact that Bidelman and colleagues used artificial sounds, iterated rippled noise (IRN). Cousineau et al. (2015) were able to replicate the effects for synthetic but not natural sounds. At the level of the auditory cortex, consonant dyads evoke larger N1m amplitudes and shorter N1m latencies than dissonant dyads (Andermann et al., 2020); remarkably, further analyses revealed even smaller differentiations between more or less dissonant dyads (Tabas et al., 2019). This effect was also seen in auditory evoked potentials (AEP) measured with electroencephalography (EEG) by Proverbio et al. (2016) but not in the results of Minati et al. (2009). Kung et al. (2014) even reported larger N1 amplitudes for dissonant intervals in musicians. An excellent summary is given in the recent review by Di Stefano et al. (2022). The above-mentioned studies all target the pitch onset response (POR) where intervals are played out of silence or noise but not in the context of a chord progression where pitch *change* responses (PCR) would be expected to arise. The sources of the POR are situated more anterior in the Heschl’s Gyrus (HG) (Krumbholz et al., 2003; Gutschalk et al., 2004).

In the visual system, posterior regions in the temporal and parietal lobe have been identified as neurofunctional correlates of gestalt perception (Bloechle et al., 2018); conversely, the term ‘gestalt’ barely appears in auditory neuroscience; moreover, consonance/dissonance has often been addressed in oddball paradigms focusing on the mismatch negativity response (MMN) (Näätänen et al., 1978). However, Park et al. (2017) explored N1m and P2m responses to chords that varied in their expectedness. Strongly expected chords went with shorter N1m/P2m latencies and larger P2m amplitudes than less expected chords. The authors concluded that P2m amplitudes might reflect the distance between chords in terms of harmonical relationships as summarized in the cycle of fifths. Cadence ending, or closure, was also part of the paradigm of Dekio-Hotta et al. (2009). A cadence leading from the dominant to the tonic in minor evoked larger N1 amplitudes than an ending on the tonic in major.

Irregular chords – no matter whether they are consonant, dissonant, or clusters – within otherwise plausible chord progressions elicit an early right anterior negativity (ERAN), independent of participant’s attention (Koelsch et al., 2000, 2007), and even if acoustical factors such as pitch repetitions or sensory dissonance were eliminated. Further studies revealed larger ERAN responses among musically trained participants depending on the degree of irregularity, resulting in larger amplitudes for greater broken expectancies (Pages-Portabella and Toro, 2020).

In general, increased musical training seems to go in line with better discrimination of tones and chords. At the brainstem level, musicians show better differentiation between different types of intervals and chords than nonmusicians (Bidelman et al., 2011a,b,c, 2014); similarly, at the cortical level, musicians were reported to have more gray matter in HG than nonmusicians (Schneider et al., 2002). Several studies also revealed greater N1(m) and P2(m) amplitudes among musicians (Shahin et al., 2003; Itoh et al., 2012; Sanju and Kumar, 2016; Andermann et al., 2021). Whereas Itoh et al. (2012) observed greater N1m amplitudes for music-experienced listeners only on the PCR, Andermann et al. (2021) found the respective difference in both the PCR and the POR.

Recent investigations by Lerousseau and Schön (2021) indicate that musical expertise is also associated with improved neural statistical learning in the auditory domain – they labeled it the *predictive coding of music model*. Studies investigating this concept use paradigms in which patterns create expectations that are violated in a few cases. Congruously, MMN responses and transient components of auditory evoked potentials/fields (AEP/AEF) such as N1(m) or even later responses from beyond the AC like N2 or P300 have been in focus. Musicians exhibit stronger MMN responses to dissonant and mistuned chords in sequences of well-tuned major chords (Brattico et al., 2009). Minor changes in sequences of dissonant intervals only evoke a late MMN in subjects with prolonged musical training (Crespo-Bojorque et al., 2018). Considering the N1m, musicians adapt more strongly to fixed pitch sequences than nonmusicians (Andermann et al., 2021). As mentioned above, the ERAN for irregular ending chord progressions is evoked in musicians as in nonmusicians, but the differentiation is more accurate and modulated by severity among nonmusicians.

Several studies have shown that some effects occur only in musicians. For example, P2 and N2 responses to intervals within an octave only match with musicological conventions in musicians (Itoh et al., 2010); similarly, N2 responses are only modulated by sensory consonance in musicians (Minati et al., 2009). Kung et al. (2014) showed that P2 and N2 amplitudes in musicians arise congruently to musicological definitions of chords as consonant and dissonant, but the responses of nonmusicians depended more on the roughness of the stimuli. Proverbio et al. (2016) detected an anterior negativity (N2) which was only enhanced in musicians in chords featuring quartertones and changes in P300 responses to frequency ranges suggesting a greater sensitivity for subtle pitch changes.

In synopsis with previous investigations, concepts like consonance and dissonance seem to be processed similarly in musicians and nonmusicians; however, it is reasonable to assume that the listener’s musicality has a great impact on neurophysiological responses and thus should be carefully controlled in auditory research.

In our study, individual musical aptitude and psychoacoustic “roundness” ratings for chord progressions were matched with auditory evoked responses measured with magnetoencephalography (MEG). MEG allows to access early auditory activity at the cortical level with superior spatiotemporal resolution (mm/ms) and high signal-to-noise ratio (SNR). In line with previous research, we hypothesized that less dissonant chords should elicit larger N1m amplitudes, in an effort to replicate previous findings in an ecologically more valid experimental design. Because dissonance can be viewed as one aspect of roundness, we further expected that if a chord progression was perceived as round then the corresponding N1m amplitude responses should also increase in amplitude. Finally, we expected that musicians would generally show larger N1m amplitudes in general, together with better discrimination of roundness.

## 2 Materials and methods

### 2.1 Participants

30 adult subjects volunteered for the experiment (15 female, 15 male, 23 right and 7 left handed). The mean age was 28.8 years (*std* = 11.3, *min* = 18, *max* = 58). None of the participants reported any hearing, neurological or psychiatric impairment. Normal hearing was verified using audiometry testing, and only participants with hearing loss <25 dB at frequencies below 4 kHz were included. All subjects provided written informed consent before participating in the experiment; moreover, in accordance with the Declaration of Helsinki, the experimental design was approved by the local ethics committee (Medical Faculty, University of Heidelberg, S-406/2021).

To measure musical aptitude, we used the Advanced Measures of Music Audiation (AMMA) (Gordon, 1989; Gordon, 1998), a test that can be completed without any musical knowledge or experience. Subjects listened to pairs of melodies and decided whether these sounded the same or varied in pitch or rhythm. A maximum score of 80 points was achievable, 40 for the tonal and rhythm part respectively; high scores reflect high musical aptitude. The overall mean was 56.2 (*std* = 8.0, *min* = 33, *max* = 73), and the median was 56. Participants were sorted into two groups along the median: high AMMA listeners and low AMMA listeners. There was no correlation between AMMA score and age (*r* = −0.082, *p* = 0.668 n.s.), no AMMA difference between male and female listeners (*t*(28) = 0.023, *p* = 0.982 n.s.), and no difference between AMMA score and age (*t*(28) = 0.446, *p* = 0.649 n.s.).

The musical background of the subjects was assessed with a questionnaire concerning their previous experience in music theory and instrumental lessons; moreover, it also included questions regarding their preferred musical genres. Six subjects reported no knowledge in music theory, 17 gained music theory lessons in school, twelve outside school, and one person had an academic degree in music. The most preferred genres were classical (20), rock (18) and pop (17). Fourteen participants declared not to make music during their free time, 15 for leisure, and one as a professional musician, but only six participants had never played an instrument in their life. Fourteen participants played at least one, ten at least two instruments. On average, participants started to take lessons on their instruments at 7.8 ± 3.3 years and continued for 9.8 ± 4.9 years. The most common instruments were piano (9), guitar (7), and violin (5).

### 2.2 Stimuli

**Figure 1:**
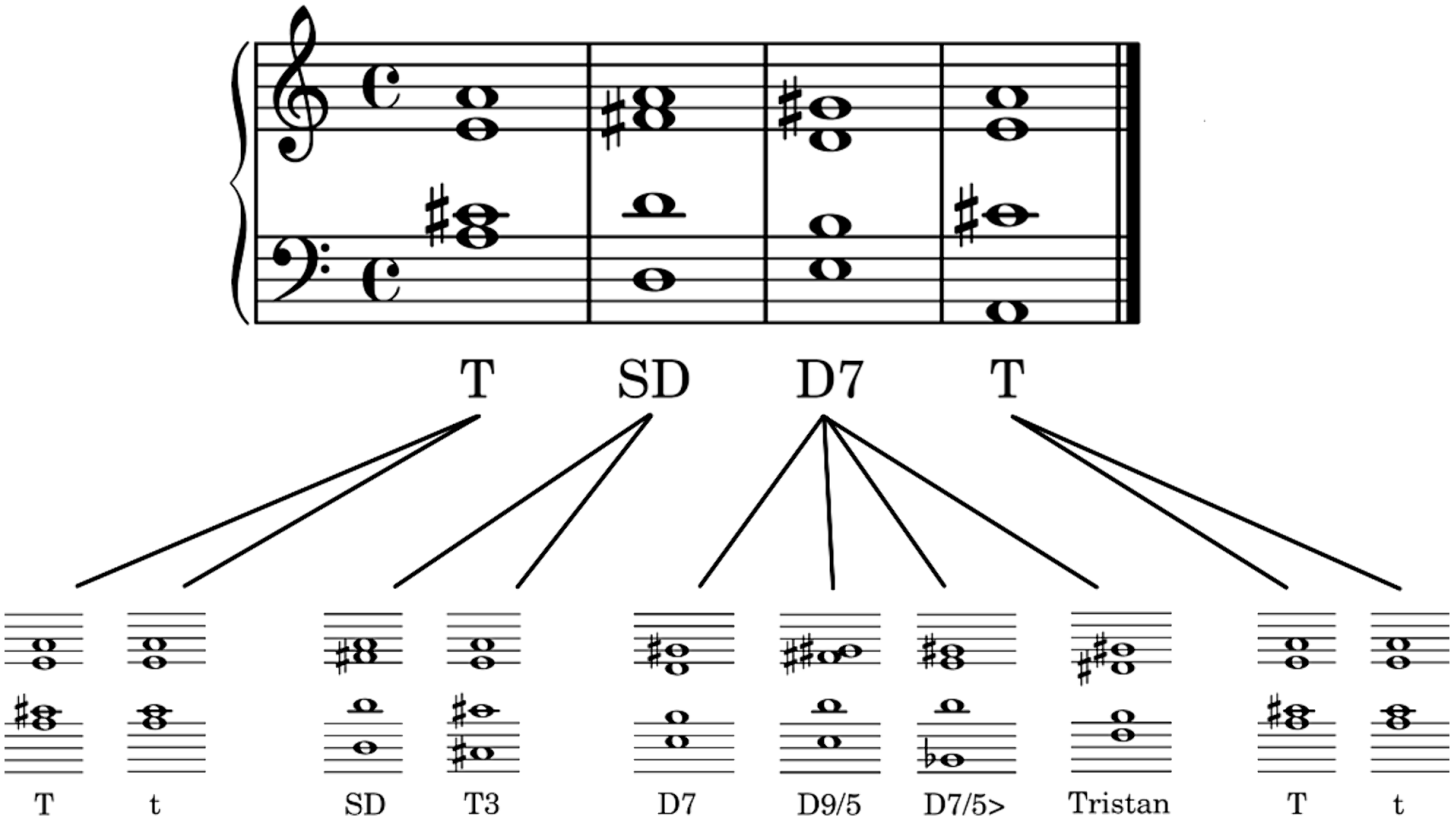
Exemplary structure of the chord progressions. There are four chords (upper row) which can be exchanged with variable elements (lower row). T, tonic major; t, tonic minor; SD, subdominant; T3, first inversion of the tonic; D7, dominant seventh; D9/5, major dominant ninth; D7/5>, dominant seventh chord with a lowered fifth (as the bass); Tristan, Tristan chord. The four chords at the third position of the sequence are listed in increasing amount of dissonance.

The stimuli were cadence-like four-chord progressions. An overview of the variable elements of the chord sequences can be seen in Figure 1. On the first chord position was the tonic, either in major (T) or minor (t), which established the tonal key and context. On the second position, it was followed either by the subdominant (SD) or the first inversion of the tonic (T3, i.e., the same tones as in the tonic, but in different order). The most prominent part of the chord progression was a dissonant dominant chord on the third position. The chord progression ended with the tonic in major or minor. All chord progressions complied with the elementary rules of western classical music theory (e.g., no parallel fifths and octaves); moreover, they were constructed such that there were no unnecessary note changes (e.g., the highest voice, on which people tend to focus most, never changed).

More specifically, at the third position of the sequence, we used four different chords from western classic music: (1) The dominant seventh chord (D7) as the most commonly used dominant chord in cadences. Although it sounds almost consonant, it contains the diminished fifth – a highly dissonant interval and often referred to as the tritone. (2) The major dominant ninth (D9/5) which is a common extension of the dominant seventh chord. (3) The dominant seventh chord with a lowered fifth (as the bass) (D7/5>) which is quite dissonant because one essential part of the basic chord (the fifth) is no longer in tune with the key. (4) The *Tristan Chord* (Tristan) which is famous for his key role in Richard Wagner’s opera *Tristan und Isolde*. It cannot be assigned to one specific musical function and became apparent for his high amount of dissonance. In musicology it is either interpreted as a secondary dominant or a subdominant chord (Danuser, 2016, ch. 2) with at least two basic notes out of key. For this experiment, we refer to the first interpretation, and we expected that the increasing degree of dissonance in the dominant chords (1)—(4) would be mirrored in the listener’s roundness ratings of the chord progressions in their entirety.

To create a great variety among the sequences, all chord progressions were transposed into two different tonal keys: *a major/minor* and *e-flat major/minor*, which are situated on opposite poles of the cycle of fifths and therefore are as far away from each other as possible in terms of tonal relationships. Three different stimulus types (or “instruments”) were used: a piano sound, which was exported from MuseScore 3 (MuseScore BVBA), an adapted cello sound from the Vienna Symphonic Library (Vienna Symphonic Library GmbH, Vienna, Austria), and artificial chords based on iterated rippled noise (IRN) (Yost, 1996) with 16 iterations. The first three chords had a duration of 750 ms, the last one was 950 ms in length to make the end of the sequence sound more naturally. Every single sequence had a total duration of 3,200 ms which was followed by a period of silence with randomly varying length between 1,300 ms and 1,350 ms. The sampling rate was 48,000 Hz, stimuli were filtered from 20 to 4,000 Hz. Each chord was equipped with hanning windows at pitch onset and offset: for the first three chords 5 and 10 ms, for the last chord 5 and 150 ms (IRN), 5 and 150 ms (piano) and 5 and 20 ms (cello). All sequences’ loudness was balanced using the integratedLoudness.m function in MATLAB (European Broadcasting European Broadcasting Union, 2014); they can be listened to at https://muwiserver.univie.ac.at/musical_roundness/.

### 2.3 MEG recordings

MEG recordings were done with a Neuromag 122-channel whole-head MEG system [Elekta Neuromag Oy, Helsinki, Finland; (Ahonen et al., 1993)], applying a sampling rate of 1,000 Hz and a lowpass filter at 330 Hz. Subjects sat inside a shielded room (IMEDCO, Hägendorf, Switzerland) and listened to the stimuli via Etymotic Research (ER3) earphones with 90 cm plastic tubes and malleable foam earpieces. Sounds were provided by a 24-bit sound card (RME ADI 8DS AD/DA interface), an attenuator (Tucker-Davis Technologies PA-5) and a headphone buffer (Tucker Davis Technologies HB-7). Using a Polhemus 3D-Space Isotrack2 system, the whole head was measured before data acquisition by registering three anatomical landmarks and 100 head surface points across the scalp, two pre-auricular points, and the nasion. This was necessary to determine the position of the head during data acquisition. While sitting in the MEG, participants watched a silent movie with subtitles to maintain stable vigilance. Subjects listened passively to the sounds in the earphones. The total recording time (88 min) was divided into two runs, offering the participants to take a break in between.

### 2.4 Psychoacoustic task

After completing the MEG measurements, subjects were asked to rate every single of the 192 chord progressions on a Likert scale from 1 to 7, considering the roundness of the whole sequence. Ratings of 1 meant *not round at all*, ratings of 7 meant *very round*. One replay was allowed until a rating was mandatory. Listeners were instructed to make their ratings based solely on their personal subjective impression.

### 2.5 MEG data analysis

MEG data were analyzed with the BESA 5.2 software package (BESA GmbH, Gräfelfing, Germany), using a spherical head model and a homogenous volume conductor. Every run of every single subject was analyzed separately. After visual inspection of the raw data, looking for noisy channels or muscular artifacts, an automatic artifact scan was run excluding all sweeps with amplitudes greater than 8,000 fT/cm or gradients greater than 800 fT/cm/ms. The epoch of every condition started 500 ms before sequence onset and lasted 5,000 ms in total. The mean activity within the last 100 ms before sequence onset were defined as the baseline. A bandpass filter of 2 to 30 Hz was used during source analysis, in which a four-dipole model on neuromagnetic responses to the second, third and fourth chord was constructed (reflecting the PCR). One pair of dipoles covered the N1m response in each hemisphere and another one the P2m response; this allowed a segregation of the different transient components. Fitting the N1m dipoles first, they were switched off to fit the P2m dipoles independently, and were then switched on again. Only dipole models with Talairach coordinates between |*x*| = 30–60, *y* = +5 – −45 and *z* = +18 – −20 were accepted. If no stable dipole model was achievable for one run (with or without including a symmetry constraint), the model of the other run was assigned. After fitting, a template source model was conducted from the averaged models of the single subject which was then automatically transferred to all single conditions using a batch script. Source waveforms were exported for every condition separately, and conditions were combined in two different paradigms: The “Basis” paradigm included the three instruments and four dissonant dominant chords; the “Cadences” paradigm included the 32 different chord progressions, pooled over instruments and tonal keys.

### 2.6 Statistical design

All statistical analyses were done with IBM SPSS Statistics, version 28.0.1.0. Psychoacoustic data were evaluated with a repeated measures analysis of variance (rmANOVA) with appropriate Greenhouse–Geisser corrections. Within-subject factors were INSTRUMENT (IRN, piano, cello), CHORD1 (major, minor), CHORD2 (SD, T3), CHORD3 (D7, D9/5, D7/5>, Tristan) and CHORD4 (major, minor); the listener’s musical aptitude was included as a between-subject factor (MUS: high vs. low AMMA listeners). Only main effects as well as first-order interactions and second-order interactions containing MUS were considered. Regarding the MEG data, separate rmANOVAs with appropriate Greenhouse–Geisser corrections were conducted on the individual listener’s mean N1m and P2m amplitudes in time windows (N1m: 30 ms, P2m: 60 ms), centered around the grand-average wave peak of each single condition, and pooled across hemispheres.

A stepwise procedure was implemented to perform the rmANOVAs on the different responses within the Basis and Cadences paradigms. In the Basis paradigm, we first performed a global rmANOVA with the factors MUS and INSTRUMENT, based on responses pooled across all four chords of the sequence (Basis I); a second rmANOVA focused solely on the third chord and included the factors INSTRUMENT, CHORD3 and MUS (Basis II). Subsequently, we turned to the Cadences paradigm where the rmANOVAs were done consecutively and based on data pooled across instruments; the factor MUS was also included in all analyses. Table 1 shows which rmANOVA factors were included at which stage of the Cadences analyses. For example, the factor CHORD1 was included in the evaluation of the neural responses to the first chord within the sequence, and so forth. If an effect reached significance, the respective factor was also included in the subsequent analysis (e.g., the factor CHORD2 in the rmANOVA of the third chord). The final rmANOVA included the factors CHORD1 and CHORD4 to check for effects of the sequence’s major/minor coherence. In all rmANOVA analyses on both neuromagnetic and psychoacoustic data, Bonferroni-correction was applied for post-hoc tests whenever *a priori* hypotheses had not been formulated.

**Table 1:** Overview of the different ANOVAs for the Cadences paradigm.

	CHORD1	CHORD2	CHORD3	CHORD4	MUS
Cadences I	X				X
Cadences II		X			X
Cadences III		X	X		X
Cadences IV			X	X	X
Cadences IV	X			X	X

Crosses mark which factor was included in which ANOVA.

The concluding MEG data analysis focused on those chord progressions which listeners had rated as particularly round or not round in the psychoacoustic task. For each participant, the 32 chord progressions were sorted according to their individual roundness ratings; then, the source waveforms of the four roundest and the four least round chord progressions were separately averaged for that participant (e.g., listener #1 judged chord progressions 1, 9, 17, and 21 as the roundest, whereas listener #2 assigned chord progressions 1, 2, 9, and 21) and fed into bootstrapping analyses (2.000 resamples), separately for the N1m and P2m amplitudes on every chord. Bootstrapping was applied instead of rmANOVA because at this granular level of analysis, it was not always possible to identify clear amplitude peaks for every individual listener and every chord progression.

## 3 Results

### 3.1 Psychoacoustic results

**Figure 2:**
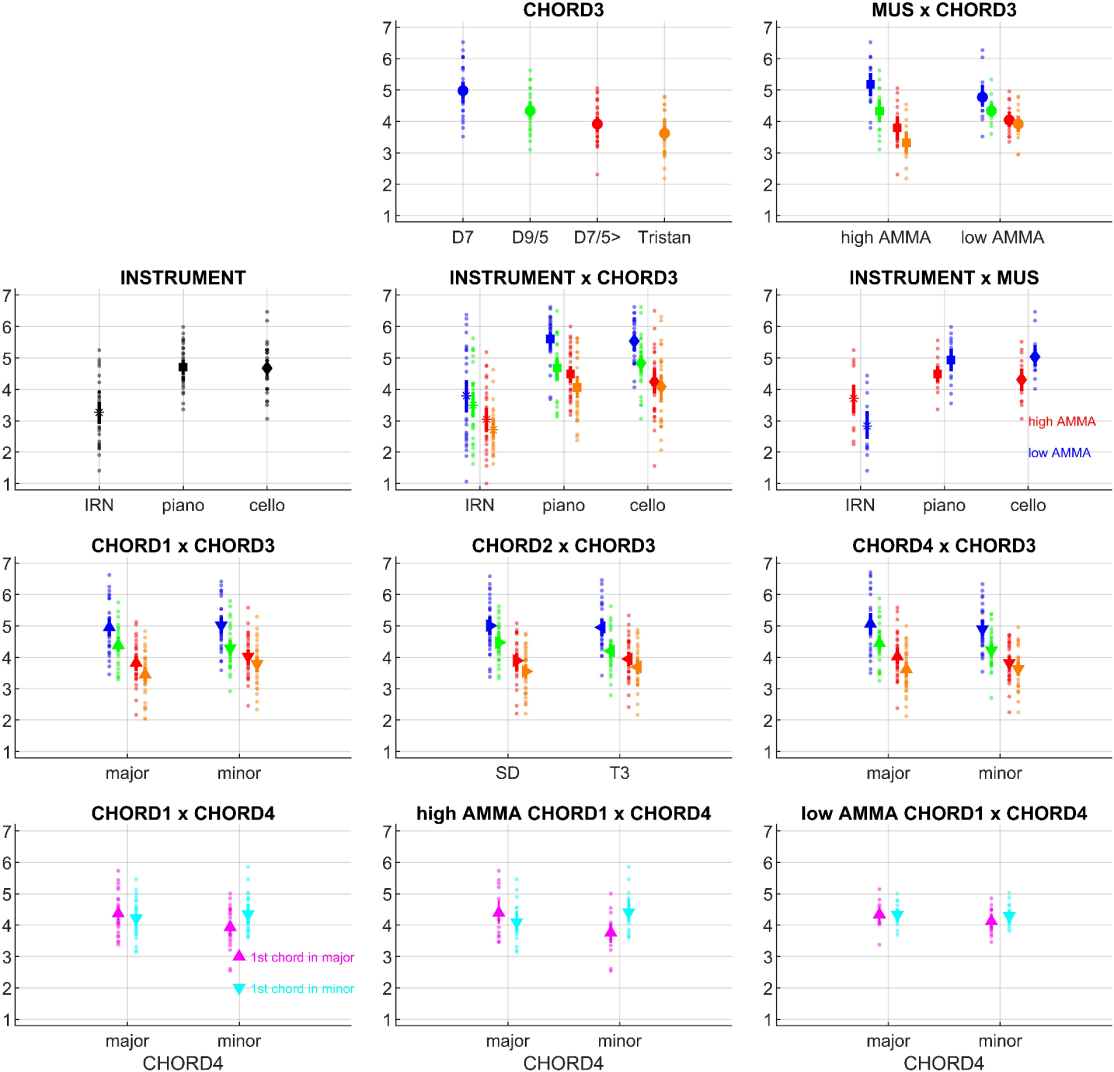
Overview of the psychoacoustic results (means and bootstrapped 95% confidence intervals), based on the data from *N* = 30 participants; small points denote individual participant’s data. Effects with *a priori* hypotheses are shown in the first row, followed by additional effects which reached significance. INSTRUMENT: iterated rippled noise (IRN), piano, cello; CHORD1: major, minor; CHORD2: subdominant, first inversion of the tonic, CHORD3: D7, dominant seventh; D9/5, major dominant ninth; D7/5>, dominant seventh chord with a lowered fifth (as the bass); Tristan, Tristan chord; CHORD4: major, minor; MUS: high AMMA, low AMMA.

For better overview, ANOVA results are reported in Table 2, significant effects are plotted in Figure 2.

**Table 2:** Psychoacoustic results.

Effect	*df*	*F*	*p*	*η_p_^2^*
INSTRUMENT	2.56	37.792	<0.001	0.574
INSTRUMENT*MUS	2.56	8.888	<0.001	0.241
CHORD1	1.28	5.214	0.030	0.157
CHORD3	3.84	40.602	<0.001	0.592
CHORD3*MUS	3.84	5.031	0.014	0.152
CHORD4	1.28	6.194	0.019	0.181
INSTRUMENT*CHORD1	2.56	7.713	0.001	0.216
INSTRUMENT*CHORD3	6.168	8.023	<0.001	0.223
CHORD1*CHORD3	3.84	5.850	0.004	0.173
CHORD2*CHORD3	3.84	10.344	<0.001	0.270
CHORD4*CHORD3	3.84	3.478	0.028	0.110
INSTRUMENT*CHORD4	2.56	10.838	<0.001	0.279
CHORD1*CHORD4	1.28	14.322	0.001	0.338
CHORD1*CHORD4*MUS	1.28	6.730	0.015	0.194

INSTRUMENT: iterated rippled noise (IRN), piano, cello; CHORD1: major, minor; CHORD2: subdominant, first inversion of the tonic, CHORD3: D7, dominant seventh; D9/5, major dominant ninth; D7/5>, dominant seventh chord with a lowered fifth (as the bass); Tristan, Tristan chord; CHORD4: major, minor; Mus: high AMMA, low AMMA; *df*, degrees of freedom; *F*, test statistics; *p*, probability value; *η_p_^2^*, partial squared eta. Aside from the results presented in this table, no further significant effects were observed at this stage of the analysis.

#### 3.1.1 Instrument, dissonance and mode influence roundness

Within-subject factors showed several significant effects. First, there were strong INSTRUMENT differences. *Post hoc* tests revealed higher roundness ratings for piano and cello sequences compared to IRN sequences (*p*’s < 0.001), whereas piano and cello sequences did not differ (*p* = 0.736). Second, the chords at position #3 within the sequence were sorted in the order D7 > D9/5 > D7/5> > Tristan according to their roundness scores; except for D7/5 > vs. Tristan, all pairwise comparisons survived *post hoc* tests (*p*’s < 0.001). Third, sequences starting in minor received higher roundness ratings than those starting in major; similarly, sequence endings in major went with lower roundness ratings than endings in minor.

#### 3.1.2 Roundness is shaped by the interplay between chords

Among the roundness ratings of the chord progressions, several interactions included CHORD3 as a factor. Regarding the CHORD1*CHORD3 interaction, D7/5 > and Tristan were associated with higher roundness ratings if a sequence started in minor (D7/5>: *p* = 0.042; Tristan: *p* = 0.008). A similar pattern occurred for the CHORD2*CHORD3 interaction where D9/5 led to higher ratings if it followed the SD and not T3 (*p* = 0.0036). The CHORD3*CHORD4 interaction appeared inverse to the above-mentioned pattern at the start of the sequence: if a sequence ended in major, D9/5 and D7/5 > went with *higher* roundness ratings (D9/5: *p* = 0.028; D7/5>: *p* = 0.048).

Aside from CHORD3-related effects, there was also an interesting CHORD1*CHORD4 interaction: When a sequence began and ended in the same, i.e., coherent mode (e.g., major on the first *and* last chord), participants assigned higher roundness ratings than for incoherent sequences (*p* = 0.008); yet, there was no difference between coherent sequences in major or minor (*p* = 0.790 n.s.). Among the incoherent sequences, the progression from minor to major was perceived as rounder than the progression from major to minor (*p* = 0.015). Finally, there was also a 2nd-order interaction of CHORD1*CHORD4 with INSTRUMENT, but this was not further analyzed due to the lack of a plausible *a priori* hypothesis.

#### 3.1.3 Musical aptitude accentuates roundness ratings

As a main effect, roundness ratings were not different between listeners with low vs. high AMMA scores; however, the between-subject factor MUS interacted with several within-subject factors. First, low and high AMMA listeners differed in how they rated sequences that were played by different instruments. In *post hoc* tests, piano and cello sequences had higher roundness scores than IRN sequences in both groups (low AMMA: *p* = 0.0018; high AMMA: *p* = 0.016), but the respective subplot in Figure 2 indicates that this difference was somewhat pronounced in low AMMA listeners. An inverse pattern emerged regarding the roundness ratings of sequences with different chords at position #3; here, high AMMA listeners showed greater rating graduations between sequences, and *post hoc* tests mirrored this effect for D7 – D9/5 (*p* = 0.023), D7 – D7/5 (*p* = 0.020), D7 – Tristan (*p* = 0.007) and D9/5 – Tristan (*p* = 0.012). Similarly, both groups rated D7 sequences rounder than Tristan sequences, but this difference was significantly stronger among high AMMA listeners (low AMMA: *p* = 0.0058; high AMMA: *p* < 0.001).

Interestingly, the above-described CHORD1*CHORD4 interaction was also influenced by MUS: *post hoc* tests showed that only high AMMA listeners distinguished significantly between coherent and incoherent progressions (low AMMA: *p* = 0.096 n.s.; high AMMA: *p* = 0.020). A differentiation within the incoherent sequences did, however, not survive *post hoc* tests in single groups (low AMMA: *p* = 0.100 n.s.; high AMMA: *p* = 0.144 n.s.), and it also did not differ between groups (*p* = 0.301 n.s.).

### 3.2 MEG results

**Figure 3:**
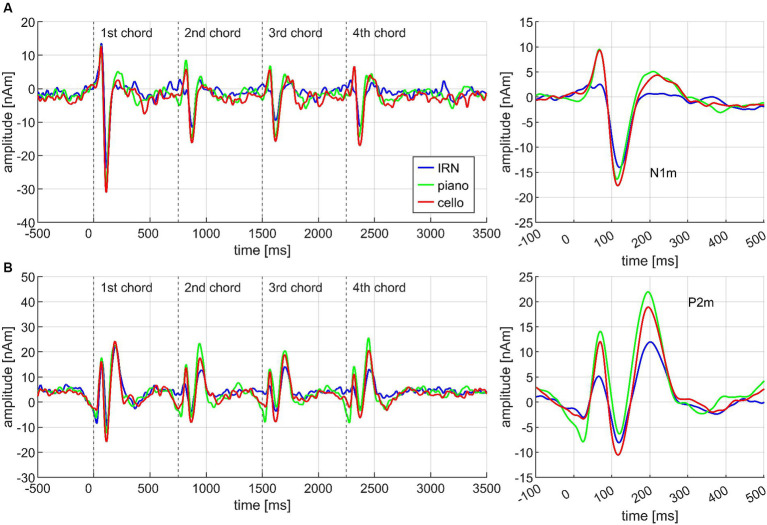
Exemplary overview of the neuromagnetic activity in response to the stimulation, based on the data from *N* = 30 participants. Each chord of the chord progression evoked a specific AEF complex which differed between chords, conditions, and instruments. The source waveforms are diagrammed separately for the N1m **(A)** and P2m **(B)** dipoles on the left side of the figure; the plots on the right side depict corresponding source waveforms, pooled over all four chords, and with additional baseline correction 100 ms before onset.

Each chord of the chord progression evoked a specific transient AEF complex which differed between chords and conditions. Figure 3 exemplarily demonstrates this for the three instruments. The source waveforms of the N1m and P2m dipoles are diagrammed separately in (A) and (B) on the left side, whereas the source waveforms pooled over all four chords and with additional baseline correction are depicted on the right side. Significant results for the Basis and the Cadences paradigm are depicted in Table 3 for better overview.

**Table 3:** MEG results.

Anova	Effect	Component	*df*	*F*	*p*	*η_p_^2^*
Basis I	INSTRUMENT	N1m	2.56	3.503	0.043	0.111
Basis I	INSTRUMENT	P2m	2.56	59.690	<0.001	0.681
Basis II	INSTRUMENT	P2m	2.56	32.786	<0.001	0.539
Basis II	CHORD3	P2m	3.84	4.318	0.010	0.134
Cadences II	CHORD2	N1m	1.28	13.598	<0.001	0.327
Cadences II	CHORD2	P2m	1.28	54.310	<0.001	0.660
Cadences III	CHORD3	P2m	3.84	5.231	0.004	0.157
Cadences III	CHORD3*CHORD2*MUS	P2m	3.84	3.231	0.030	0.103
Cadences IV	CHORD3	N1m	3.84	3.159	0.031	0.101
Cadences IV	CHORD3*MUS	N1m	3.84	2.933	0.041	0.095
Cadences IV	CHORD3*CHORD4	P2m	3.84	3.733	0.016	0.118
Cadences V	CHORD1*CHORD4	P2m	1.28	9.307	0.005	0.249

Basis I: repeated measures ANOVA on Basis paradigm pooled over all chords (INSTRUMENT, CHORD3, MUS); Basis II: repeated measures ANOVA on Basis paradigm on third chord position (INSTRUMENT, CHORD3, MUS); Cadences II: repeated measures ANOVA on Cadences paradigm on the second chord position (CHORD2, MUS); Cadences III: repeated measures ANOVA on the third chord position (CHORD2, CHORD3, MUS); Cadences IV: repeated measures ANOVA on fourth chord position (CHORD3, CHORD4, MUS); Cadences V: repeated measures ANOVA on forth chord position (CHORD1, CHORD4, MUS); Instr: instrument, iterated rippled noise (IRN), piano, cello; CHORD1: major, minor; CHORD2: subdominant, first inversion of the tonic, CHORD3: D7, dominant seventh; D9/5, major dominant ninth; D7/5>, dominant seventh chord with a lowered fifth (as the bass); Tristan, Tristan chord; CHORD4: major, minor; Mus: high AMMA, low AMMA; *df*, degrees of freedom; *F*, test statistics; *p*, probability value; *η_p_^2^*, partial squared eta. Aside from the results presented in this table, no further significant effects were observed at this stage of the MEG data analysis.

#### 3.2.1 Cello and piano elicit larger AEF amplitudes than IRN

**Figure 4:**
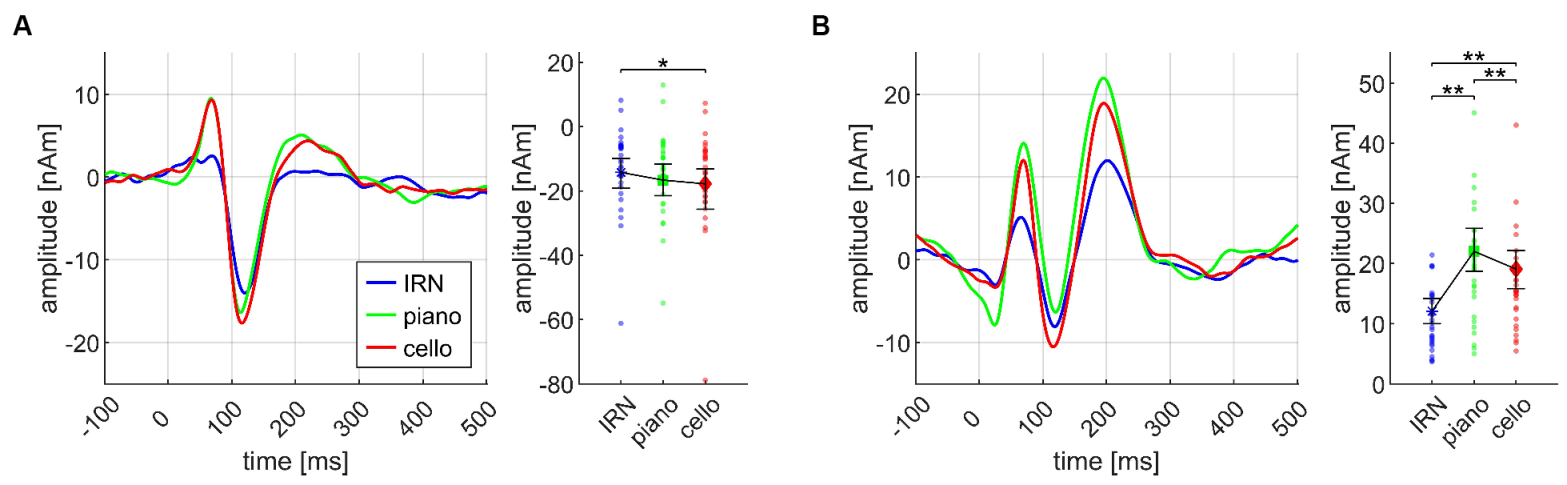
Transient components evoked by different instruments (IRN, piano, cello), pooled over all chords (Basis I), shown separately for the N1m **(A)** and the P2m **(B)** dipoles, and based on the data from *N* = 30 participants. In both panels, source waveforms are shown together with their corresponding 95% bootstrap confidence intervals; small points denote individual participant’s data. *p < 0.05, **p < 0.001.

ANOVA on the Basis paradigm showed significant INSTRUMENT effects for both N1m and P2m (see Figure 4). *Post hoc* tests ensured larger N1m amplitudes for cello than IRN (*p* = 0.018). Piano elicited larger P2m amplitudes than IRN and cello (*p*’s = 0.0027) and cello than IRN (*p* = 0.0018). The P2m effect also remained in the Basis II ANOVA which was exclusively based on the third chord. Here, after *post hoc* tests, cello and piano still evoked larger P2m amplitudes than IRN (each *p* = 0.0029), and piano compared to cello (*p* = 0.019).

**Figure 5:**
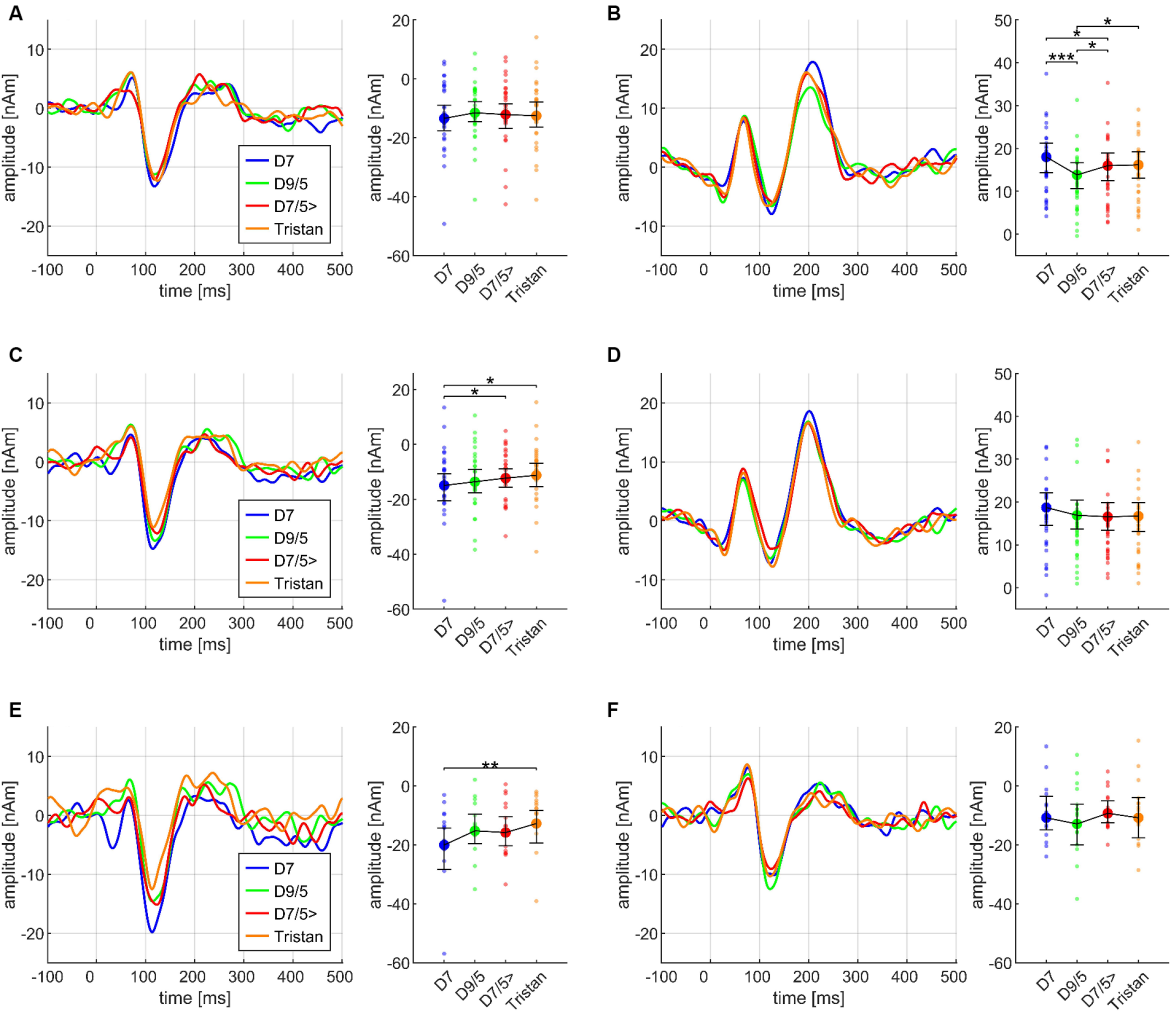
Transient components in response to the dissonant chords (CHORD3: D7, dominant seventh; D9/5, major dominant ninth; D7/5>, dominant seventh chord with a lowered fifth (as the bass); Tristan, Tristan chord) on the third and fourth chord position within the sequence, based on the data from *N* = 30 participants. In each subplot, the source waveforms are presented together with their corresponding 95% bootstrap confidence intervals, separately for N1m **(A)** and P2m **(B)** on the third chord position, and N1m **(C)** and P2m **(D)** on the fourth chord position; small points denote individual participant’s data. N1m responses differed between listeners with high **(E)** and low **(F)** AMMA scores. *p < 0.05, **p < 0.001, ***p < 0.001; n.s., not significant.

Regarding CHORD3, P2m differences reached significance in the Basis paradigm, but *post hoc* test revealed that this effect was mainly driven by the difference between D7 and D9/5 (*p* < 0.001), with greater amplitudes for D7, along with greater amplitudes for D7/5 > compared to D9/5 (*p* = 0.033) and greater amplitudes for Tristan compared to D9/5 (*p* = 0.048). The direction of the latter two comparisons did not match our *a priori* postulated hypotheses (greater amplitudes for D9/5 than D7/5 > and Tristan). The identical effect re-occurs in the Cadences paradigm (Cadences III) and will be illustrated in Figure 5B. Importantly, there was no INSTRUMENT*CHORD3 interaction (*F*(6,168) = 0.699, *p* = 0.578, n.s.) which allowed us to pool across instruments in subsequent analyses. The factor MUS did not yield any significant main or interaction effects within the Basis paradigm.

#### 3.2.2 CHORD3 shapes N1m at the *fourth* CHORD, accentedly in high AMMA listeners

**Figure 6:**
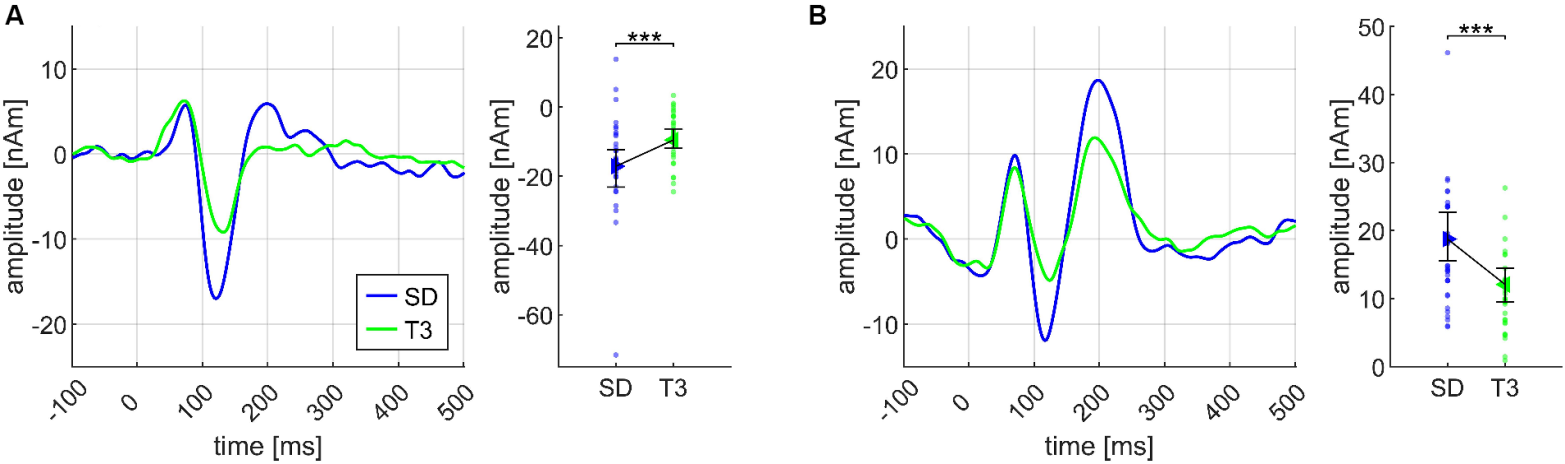
Transient components evoked by the second chord (CHORD2: subdominant, first inversion of the tonic), shown separately for the N1m **(A)** and the P2m **(B)** dipoles, and based on the data from *N* = 30 participants. In both panels, source waveforms are shown together with their corresponding 95% bootstrap confidence intervals; small points denote individual participant’s data. ***p < 0.001.

Regarding the second chord in the sequence, both N1m and P2m showed significantly larger amplitudes for SD than for T3 (Cadences II; see Figure 6). At the third chord (Figure 5), there was no CHORD3 main effect for the N1m; however, significant P2m differences occurred in the same manner as in the Basis II ANOVA, consisting of the difference between D7 and D9/5 > (*p* < 0.001), D7 with greater amplitudes than D7/5 (*p* = 0.023), D 9/5 with smaller amplitudes than D7/5 > (*p* = 0.034), and D9/5 with smaller amplitudes than Tristan (*p* = 0.024) (Cadences III, see Figure 5B). P2m analyses also revealed a significant CHORD2*CHORD3*MUS interaction, but since the CHORD2 main effect was not significant on the third chord position and *post hoc* tests did not lead the way for further insights, this effect was treated as less relevant.

Importantly, however, CHORD3 had a significant main effect on the N1m amplitude at the *fourth* chord position (Cadences IV, *cf.* Figure 5C). *Post hoc* test revealed greater amplitudes for D7 compared to Tristan (*p* = 0.003) and D7/5 > (*p* = 0.012). The order of amplitudes confirmed the *a priori* hypothesis D7 > D9/5 > D7/5> > Tristan. The factor MUS also had a significant impact on this effect: N1m amplitudes differed significantly between D7 and Tristan for high AMMA listeners (*p* = 0.004) but not for low AMMA listeners (*p* = 0.171 n.s.). The order of the amplitudes in the high AMMA, but not in the low AMMA group matched musicological expectations (*cf.* panels E and F in Figure 5). The CHORD3*CHORD4 interaction was significant among the P2m amplitudes at the fourth chord position, but again *post hoc* tests did not show any meaningful pattern.

#### 3.2.3 Mode coherence is reflected in the P2m amplitude

**Figure 7:**
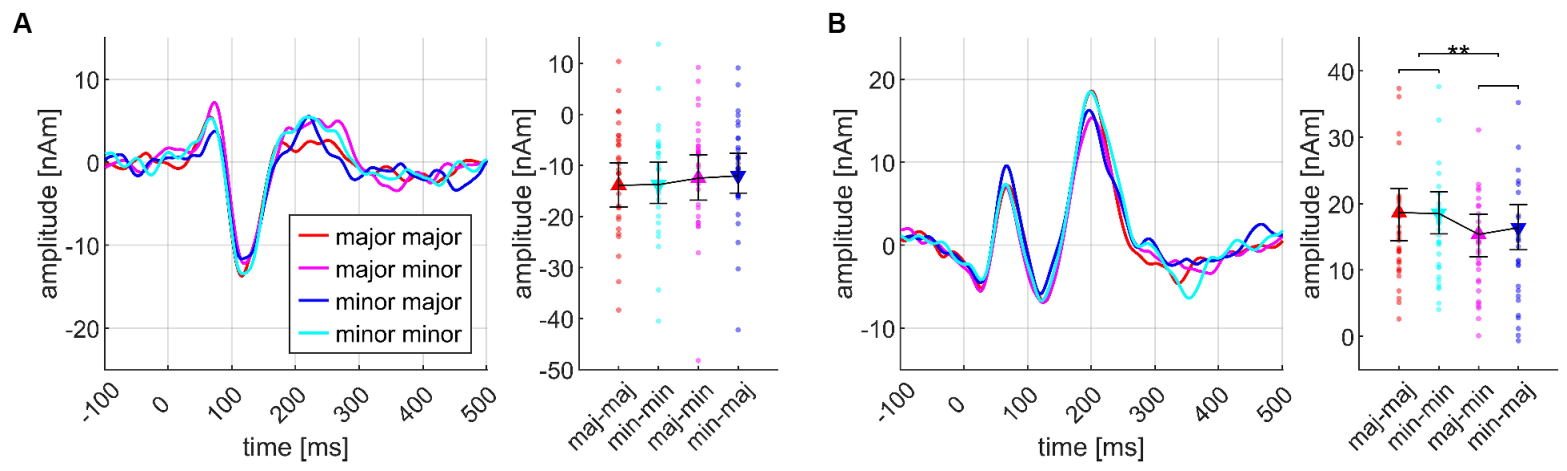
Transient components evoked at the fourth chord position, depicting the interaction effect between CHORD1 (major, maj, minor, min) and CHORD4 (major, minor), based on the data from *N* = 30 participants. In each subplot, the source waveforms are presented together with their corresponding 95% bootstrap confidence intervals, separately for N1m **(A)** and P2m **(B)**; small points denote individual participant’s data. **p < 0.001.

Although CHORD1 and CHORD4 were no significant main effects at their respective chord positions, the psychoacoustic results reported above pointed out an effect of major/minor coherence during the course of the chord sequence. Indeed, at the fourth chord position, an ANOVA with both factors revealed a highly significant CHORD1*CHORD4 interaction (Cadences V, see Figure 7). Here, N1m and P2m amplitudes both showed a comparable amplitude pattern, but only the P2m amplitude was significantly modulated by the coherence of the sequence. *Post hoc* tests revealed greater P2m amplitudes for coherent sequences (major-major, minor-minor) than for incoherent sequences (major-minor, minor-major; *p* = 0.008), whereas the two incoherent sequences did not differ (*p* = 0.774 n.s.).

#### 3.2.4 N1m mirrors roundness evolution during chord progressions

**Figure 8:**
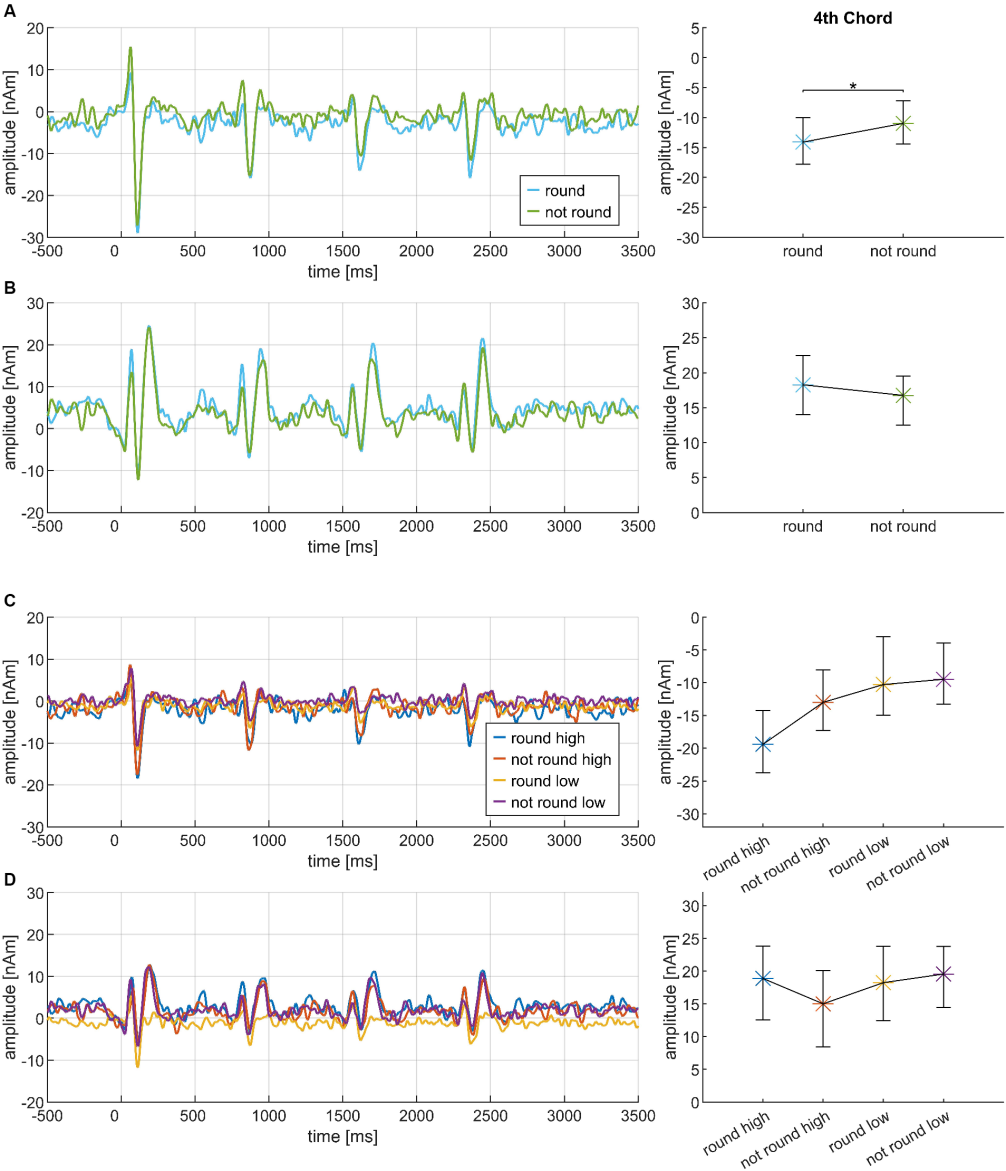
Transient components, sorted according to the individual roundness ratings of the four highest (round) and lowest (not round) rated chord progressions, shown separately for N1m **(A)** and P2m **(B)** and based on the data from *N* = 30 participants. Each panel presents the source waveforms along the sequence, together with their corresponding 95% bootstrap confidence intervals at the fourth chord. Panels **(C,D)** of the figure are organized in a similar manner, but present the data separately for listeners with low vs. high AMMA scores (high, high AMMA; low, low AMMA). *p < 0.05.

Figure 8 presents a comparison of the neuromagnetic responses to those sequences which had been rated as particularly round vs. unround by the listeners; here, we present the corresponding sequences grouped according to the ratings of *individual* listeners. Note the dynamic changes of the components: The first two chords show no differences in N1m amplitudes, begin to segregate on the third chord, and reach the maximal difference on the fourth chord. However, the P2m amplitudes start segregating on the second chord (where psychoacoustics did not show a roundness difference), continuing to the third chord, but diminishing on the fourth chord. Ratings were given by the participants after the last chord. Therefore, it seems more reasonable to assume that correlates of roundness might be reflected in N1m rather than P2m amplitudes. High AMMA listeners tended to show greater differences in their N1m amplitudes between round and not round sequences, but this effect just missed significance (*p* = 0.0615 n.s.).

## 4 Discussion

To our knowledge, this study is the first to investigate the neuromagnetic representation of musical roundness. Our experimental design embeds the concept of consonance/dissonance (CD) in a broader context of chord progressions, grounded on Western music theory; and we investigated listener’s apprehension of these chord progressions, *as a whole*, including both neuromagnetic and psychoacoustic measures, and taking into account the listener’s individual musicality. In the remainder of this work, we will summarize and discuss our findings with their relations to existing and implications for future work.

### 4.1 Consonance/dissonance

Regarding the chords at the third position of the chord progression, their gradation in dissonance could be convincingly demonstrated in the psychoacoustic task: Both high and low AMMA listener rated the chords in the predicted order, with decreasing scores from the D7 to the Tristan chord. This result is in line with Arthurs et al. (2017) finding that more often used chords are perceived as more consonant. However, contrary to our expectations, there were no differences in the N1m responses to these chords. This is at odds with results at subcortical (Bidelman and Krishnan, 2009; Bidelman and Krishnan, 2011; Bidelman, 2013; Bidelman and Grall, 2014) and cortical levels (Andermann et al., 2020) which point to larger responses for consonant intervals. Up to date, representations of CD in the auditory cortex, especially in the N1(m) wave, have not been fully understood; moreover, our paradigm differs from previous designs in two important aspects: Firstly, the chords in our experiment were played in the greater musical context of a chord progression and not out of silence or noise. Therefore, the neural response did not capture the POR, but the PCR. Secondly, our paradigm used diverse sound classes instead of only one stimulus type. At the brainstem level, Cousineau et al. (2015) could reproduce Bidelman and Krishnan (2011) findings only for synthetic but not for natural sounds. Minati et al. (2009) used natural piano sounds and did not see differential effect in evoked EEG responses, whereas Proverbio et al. (2016) and Kung et al. (2014) used sinus wave tones and reported contrary findings (Di Stefano et al., 2022). Our study contributes to this heterogeneous pattern by presenting a four-dipole model based on source analyses of MEG data. The model allowed for a clearer separation of N1m and P2m activity, in contrast to the two-dipole model that was used by Andermann et al. (2020).

To our surprise, the N1m amplitude differences that were expected to arise after the third chord did, in fact, occur in the responses to the *fourth* (i.e., the final) chord of the sequence: If a D7 was played directly before, the N1m response to the fourth chord was larger than to the other chords, with decreasing amplitudes for decreasing roundness ratings. One might argue that this view is problematic because here listeners did not hear the dissonant chords itself but its subsequent resolution. However, we think this approach is decent because the psychoacoustic ratings were also given after the whole sequence was played. A possible reason for this delayed representation of the dissonant chords could be that neuromagnetic CD correlates emerge differently if a broader musical context is available; then, the PCR would not solely reflect “absolute” CD, unlike the POR at sequence onset. In this sense, the chord’s absolute dissonance would be less relevant than the relationship between subsequent chords in a progression. A similar phenomenon has recently been observed by Andermann et al. (2021) regarding single pitch; here, the POR mirrored the absolute pitch value whereas the PCR reflected relative changes in pitch. Transferred to the current experiment, the Tristan chord would only unfold its full dissonance in relation to more consonant chords. This interpretation, however, neglects that the chord preceding the Tristan chord was *always* consonant (either SD or T3), i.e., there was a substantial increase of dissonance from chord two to chord three. Another, related explanation for the above-described N1m response pattern might be that it is not a correlate of CD itself but of a related concept which might be described by the term *roundness.* Roundness can be understood as an aspect of gestalt that evolves over time. This view of CD and roundness is linked to Terhardt’s understanding of CD as sensory consonance and harmony. If the POR of a single, isolated chord is recorded, then sensory consonance can be expected to shape the waveform morphology. However, if the chord lines up with other chords, then harmony becomes more important and would be expected to shape the PCR. The current study is not able to fully disentangle the PCR to consonant and dissonant chords in this context; future studies are needed that explicitly target the contrast between sensory CD and harmony.

### 4.2 Musical context/roundness

In our study, listeners rated chord progressions as rounder when they were made from piano or cello rather than from IRN sounds. This goes in line with previous findings that familiar timbre lets chords appear more consonant. However, the corresponding N1m/P2m amplitude differences are most likely due to divergent physical characteristics of the sounds. IRN has spectral maxima at the multiples of the fundamental frequency, but its noisy spectrum does not contain formants like piano and cello sounds. N1m amplitudes are known to be larger for non-linear, natural sounds than for linear stimuli (Mizuochi et al., 2005); e.g., piano sounds elicit larger N1m amplitudes than noise or pure tones (Lütkenhöner et al., 2006).

Although the psychoacoustic ratings did not reveal differences between SD and T3 at the second chord in the sequence, the corresponding N1m and P2m amplitudes differed strongly, with much larger amplitudes for SD. The most probable explanation for this effect is adaptation. If a stimulus is repeated, the response to the second stimulus is smaller in magnitude. This is true for single notes (Patterson et al., 2016; Andermann et al., 2021) as well as for chords following other chords (Park et al., 2017) or scales (Otsuka et al., 2008). Similar to Park et al. (2017), the second chord in our study shows an adaption which likely relates to the harmonical context: The first inversion of the tonic is not the exact same chord, but its function in the musical context is the same.

Roundness ratings also differed between major vs. minor at the first and last chords of the sequence. Chord progressions starting in minor were judged as rounder than those starting in major; conversely, when chord progressions ended in minor, they were rated as less round than those ending in major. This, however, was not reflected in the neuromagnetic data, and there were no mode-related *a priori* hypotheses; thus, interpretation is challenging and interaction effects are likely the main drivers at this point. Regarding the first chord, one could argue that roundness ratings in minor-starting chord progressions were slightly higher if they contained the Tristan chord, which might have influenced the main effect. Regarding the last chord, it is crucial to consider the interaction between the first and the last chord of the sequence: Mode-coherent chord progressions led to higher roundness ratings than incoherent chord progressions. Comparing the two mode-incoherent chord progressions, listeners perceived the minor-major transition as rounder than the major-minor transition. This may explain the significant main effect among the fourth chord. Furthermore, it refers to the convention of the Picardy third era (i.e., the use of the major third in an otherwise minor musical context) from the 16th century until the baroque, a popular stylistic device to create a stronger impression of closure (Rushton et al., 2001, para. 1). Furthermore, mode-coherent chord progressions evoked larger P2m amplitudes in our experiment. Park et al. (2017) proposed an inverse relationship between P2m amplitudes and harmonic distance, with larger P2m amplitudes for more closely related and expected chords. Transferred to our findings, this would explain why mode-incoherent chord progressions elicited smaller P2m amplitudes because the major vs. minor realizations of a chord are rather distant in terms of their harmonic relationship. Another explanation is that the larger P2m amplitudes in mode-coherent progressions are the result of higher expectancy; however, Park et al. (2017) actually noticed better reflection of such expectations in the P2m latency. In sum, both approaches provide only poor explanations for the observed effects. Chord progressions from a major tonic over the dominant to a minor tonic elicit larger N1 amplitudes than major tonic endings (Dekio-Hotta et al., 2009). It might be the case that both effects overlapped so that none of them got significantly established. Considering the tendencies, they showed higher N1m amplitudes for rounder closings.

The evolution of N1m/P2m amplitudes along several chords provides new insights into roundness perception over time. At the first two chords, N1m amplitudes did not differ between sequences which the listeners rated as round vs. unround. Starting with the third chord, however, there evolves an N1m difference that reaches significance at the fourth chord, with larger N1m amplitudes in chord progressions that were judged as rounder. This observation nicely conforms with the *a priori* hypothesis, and it seems reasonable to argue that roundness perception is associated with N1m dynamics during the emergence of an acoustic gestalt. We assume this finding to be separable from ERAN or MMN activity. ERAN can indeed influence N1 amplitudes (Sauvé et al., 2021), but our study did not manipulate musical syntax or violate respective expectations. Moreover, explaining our findings with ERAN or MMN is hardly feasible because we did not see larger N1m responses to less round chords and chord progressions; in fact, we found that round chords and chord progressions went with larger N1m amplitudes. In turn, the P2m amplitude dynamics are more difficult to interpret; given the observed response pattern, it does not seem plausible that this wave acts as a direct neural correlate of musical roundness.

We should concede that most of the roundness-associated effects have smaller effect sizes than the T3 vs. SD adaptation effect at the second chord or the differences related to sound type (Table 3). It is possible that the high ecological validity of our experimental design might also increase the risk of interference between CD, roundness and concepts like adaptation or stimulus timbre. Remarkably, there are N1m patterns in our data that cannot be explained with previous approaches like ERAN or MMN. One could argue that the definition of roundness allows too much for different interpretations, but the clear psychoacoustic results convincingly demonstrate that our participants understood the term in a highly similar way. Further, N1m amplitude differences need not necessarily be caused by processing in the auditory cortex only: Oscillatory generators or coupled brain areas could also lead to such differences or interfere with AC generators (Park et al., 2011). The N1 wave then should be viewed more as a mirror of neural processing in higher-level areas; this claim, however, certainly warrants further research.

We would like to conclude this subsection by directing the reader’s attention to some historical references that nicely illustrate the importance of roundness in music and its conceptual proximity to gestalt. In the 19th century, musical roundness (German: ‘Rundung’ or ‘Geschlossenheit’) had the meaning of a fine and even playing style (e.g., Schubart, 1806, p. 75: ‘welche Harmonie, welche Rundung des Vortrags, welche Einheit, welcher Tonflug!’ [‘What harmony, what roundness of the performance, what unity, what flight of sound!’]). From Paul Hindemith’s ‘Übungsbuch für den zweistimmigen Satz’ ([Exercise book for the two-part movement], 1939) onwards, the term roundness was used for chord progressions that return to their beginning (Hindemith, 1939, p. 18: ‘Indem wir zum Schlüsse an den Ausgangspunkt zurückkehren, erzielen wir beim Hörer das Gefühl formaler und tonaler Rundung und Geschlossenheit.’ [‘By returning to the starting point at the end, we achieve a feeling of formal and tonal roundness and unity in the listener’], similar: p. 124). In the same sense, roundness can be found, for example, in Ernst Kurth’s (1956) widely used ‘Grundlagen des linearen Kontrapunkts’ ([Basics of linear counterpoint], e.g., from p. 150–180 on almost every page). To sum up, musical roundness might help to reframe the above-discussed consonance/dissonance aspect to a more holistic, gestalt-like understanding that covers the relations between musical chords.

### 4.3 Musicality

In our experiment, there was only a tendency of larger neural activity among high AMMA listeners, but there was no global effect regarding N1m/P2m amplitudes, in contrast to previous findings on the POR (Shahin et al., 2003; Andermann et al., 2021) and PCR (Itoh et al., 2012; Andermann et al., 2021). Andermann et al. (2021) demonstrated musicality effects in both the POR and the PCR using sequences of single IRN stimuli instead of chords or natural instruments. One could think that the use of various sound classes might have prevented the musicality effect from reaching significance; on the other hand, both high and low AMMA listeners have no experience with an artificial stimulus like IRN in their every-day lives. Further studies are needed to explore this discrepancy.

High AMMA listeners showed greater differences in their roundness ratings regarding the mode-coherence of chord progressions, and also regarding the four dissonant chords at the third position of the sequence; moreover, their ratings appeared somewhat less different between instruments, i.e., IRN vs. piano/cello. The order of dissonant chords was also reflected in the N1m responses to the fourth chord. This is reminiscent of earlier research confirming more precise representations of auditory evoked responses in musicians (Ahonen et al., 1993; Park et al., 2017; Pages-Portabella and Toro, 2020). However, Linnavalli et al. (2020) could only demonstrate an impact of musicality on psychoacoustic data but not on MMN responses. In their study, subjects were asked to decide which of two chords was more dissonant, and musicians showed greater accuracy but did not differ from non-musicians in their neural activity. The authors assumed that (sensory) consonance processing is a general aspect that does not need further training. Those findings do not necessarily stand in contrast with our results; moreover, sample characteristics were also different between Linnavalli et al. (2020) and our study. It could be the case that differences in musicality become more important with increasing complexity of the auditory stimulation. This interpretation is similar to the work of Crespo-Bojorque et al. (2018) who showed that musicians have an advantage in detecting changes in dissonant, but not consonant interval sequences.

Our finding that high AMMA listeners had larger rating differences between the dissonant chords reminds of the finding that musically trained individuals show a greater range of consonance ratings (Arthurs et al., 2017). These authors speculated that musicians perceive dissonant chords as more dissonant than non-musicians because they know about their meaning for tonal hierarchy. In a similar vein, one could also argue that the low AMMA listeners might have misunderstood the term *roundness* itself; however, although we cannot rule out this objection completely, one can strongly assume that that the clear psychoacoustic results for the dissonant chords can be a proxy that all participants had a similar concept in mind. Similarly, despite the fact that we found a significant interaction between dissonant chords and instruments, the order of the dissonant chords always remained the same, and it merely seemed to be easier to detect differences between the chords if the stimulus was a cello or piano sound which goes in line with work of Lahdelma and Eerola (2020).

### 4.4 Strengths and limitations

This study adds to existing research in a number of ways. The sound sequences in our experiment closely follow Western music theory and provide a valuable approximation to real-world music with its contextualized interplay of consonant and dissonant chords. Unlike Sauvé et al. (2021) who struggled to interpret their results because of confounding pitch changes, this aspect was controlled in the current paradigm where the highest voice was kept constant. Moreover, psychoacoustic ratings targeted chord progressions instead of single chords, allowing the gestalt concept to enter CD perception. As a further advantage, the inclusion of both artificial and naturalistic sounds allows use to draw conclusions beyond a specific stimulus type, and the role of the listeners musicality was explicitly assessed as an experimental factor. Regarding the MEG data, the four-dipole model developed by spatiotemporal source analysis enabled an accurate division of transient N1m/P2m components; and the functional pattern of these components differs from other prominent neural responses because in the current experiment, neither was musical syntax hurt (ERAN) nor were expectancies broken systematically (MMN). Our results further corroborate the advantages of MEG in assessing early auditory processing at the cortical level; specifically, its combination of excellent spatiotemporal resolution and high SNR represents an attractive way to study the cortical foundations for music perception in the brain.

Despite its high flexibility and ecological validity, some limitations come with this study. Initially, it was planned to include latency analyses of the neuromagnetic responses to the chord progressions, since consonant chords are known to elicit N1m waves with shorter latency than dissonant chords (Tabas et al., 2019; Andermann et al., 2020). However, in the current study, identification of clear peak latencies was not always possible for every single listener, condition and component/dipole, due to the heterogeneous stimulus set; as a consequence, we had to limit analyses to a mean-amplitude measure. As a second aspect, the vast majority of participants in our study were born in Germany and had lived there for at least several years, in which they were frequently exposed to Western music. Judgments on CD and chord preferences do not only depend on acoustic characteristics but also on individual aspects such as preferred genres (Popescu et al., 2019) and cultural familiarity (Lahdelma and Eerola, 2020). Our study explicitly tried to bypass this by avoiding terms like consonance/dissonance or asking for rule violations in Western music theory. Still, to confirm the findings of this study, the roundness concept should be also transferred to other (both Western and non-Western) music styles like jazz or Indian raga. Moreover, it would be advisable for future studies on roundness to overcome the customary ‘musician’ vs. ‘nonmusician’ dichotomy and broaden one’s view to other listener groups like, e.g., children, adolescents, musicologists or composers; here, again, cross-cultural comparisons would certainly be enriching and might perhaps even affect music education.

## 5 Conclusion

In summary, this study emphasizes that experimental designs which mimic real-world music are valuable for the investigation of auditory perception and its neural correlates. Consonance and dissonance seem not to be reflected in the N1m amplitude as an absolute value but rather depend on the musical context. N1m amplitudes increase in magnitude when a chord (progression) is perceived as round. Whether this has its origin in the N1m generators themselves or in top-down influences from higher brain regions warrants further study. Listeners with greater musical aptitude seem to have a more distinct representation of roundness in terms of psychoacoustics and, partly, their neuromagnetic responses.

## Data availability statement

The raw data supporting the conclusions of this article will be made available by the authors, without undue reservation.

## Ethics statement

The studies involving humans were approved by the Ethics Committee of the Medical Faculty, University of Heidelberg (Alte Glockengießerei 11/1, 69115 Heidelberg, Germany). The studies were conducted in accordance with the local legislation and institutional requirements. The participants provided their written informed consent to participate in this study.

## Author contributions

SW: Conceptualization, Data curation, Formal analysis, Investigation, Methodology, Visualization, Writing – original draft, Writing – review & editing. CR: Conceptualization, Methodology, Visualization, Writing – review & editing. AR: Conceptualization, Formal analysis, Project administration, Resources, Supervision, Validation, Writing – review & editing. MA: Conceptualization, Data curation, Formal analysis, Methodology, Software, Supervision, Validation, Visualization, Writing – review & editing.
